# Toxicological Evaluation of Low Molecular Weight Fucoidan in Vitro and in Vivo

**DOI:** 10.3390/md14070121

**Published:** 2016-06-24

**Authors:** Pai-An Hwang, Ming-De Yan, Hong-Ting Victor Lin, Kuan-Lun Li, Yen-Chang Lin

**Affiliations:** 1 Department of Bioscience and Biotechnology, National Taiwan Ocean University, Keelung 20246, Taiwan; amperehwang@gmail.com; 2 Cancer Center, Wan Fang Hospital, Taipei Medical University, Taipei 11696, Taiwan; yanmd717@gmail.com; 3 Department of Food Science, National Taiwan Ocean University, Keelung 20246, Taiwan; HL358@ntou.edu.tw; 4 Graduate Institute of Biotechnology, Chinese Culture University, Taipei 11114, Taiwan; noodleplusalan@gmail.com

**Keywords:** low molecular weight fucoidan, toxicity, *Laminariajaponica*

## Abstract

For a long time, fucoidan has been well known for its pharmacological activities, and recently low molecular weight fucoidan (LMF) has been used in food supplements and pharmaceutical products. In the present study, LMF was extracted from *Laminaria* *japonica* by enzyme hydrolysis. The toxicity of LMF in mouse and rat models was determined by many methods, such as total arsenic content, bacterial reverse mutation assay, chromosome aberration assay, and in vivo micronucleus assay. The present findings showed that LMF at 5000 μg/mL exhibited no mutagenicity. It also produced no formatting disruption of red blood cells in vivo. At 2000 mg/kg BW/day there were no toxicological indications. LMF is expected to be used as a safe food supplement.

## 1. Introduction

Prior to the 1950s, seaweeds were used as traditional and folk medicines [1]. Biologically active compounds from brown seaweed, however, were not discovered until the 1990s. Fucoidan is the general term for a class of sulfated and fucosylated polysaccharides found in brown seaweed; it was identified by Kylin [2]. The intensity of fucoidan’s biological activities varies with species, molecular weight, composition, structure, and method of extraction [3], and its non-animal origin has been related to particular pharmacological activities [4]. Fucoidan has been well studied concerning its antitumor [5,6,7], antiviral [8], anti-inflammatory [9,10], anticoagulant [11], and osteogenic-enhancing differentiation activities [12]. Those activities, however, are closely related to molecular weight [13] and sulfate content [14]. Low molecular weight fucoidan (LMF) shows greater potency in its bioactivities than does high molecular weight fucoidan (HMF) [15]. Previous studies have demonstrated that LMF has high bioactivities in vitro and in vivo [5,6,7,12]. In this study, LMF has therefore been investigated for its toxicity at various concentrations.

*Laminaria japonica* is known as sea kelp and is one of the popular edible brown seaweeds in many countries. In recent years, *L. japonica* has been cultured extensively and different strains have been bred to improve its production for commercial use [16]. Fucoidan extracted from *L. japonica* has been studied extensively for its diverse biological activities [17]. Only Li et al., however, have reported on its acute and sub-chronic toxicity [18]. Furthermore, brown seaweed accumulates arsenic (As) during its growth and its total arsenic content is relatively higher than that of green or red seaweed [19]. The As compounds in brown seaweed include major organic forms, such as monomethylarsonic acid (MMA), dimethlarsinic acid (DMA), arsenobetaine (AsB), and arsenocholine (AsC). These are significantly less toxic than inorganic forms, such as arsenite (AsIII) and arsenate (AsV) [20,21]. Organic arsenic, however, can be a cancer promoter [22].

The demand for fucoidan has increased, driven by its use in food supplements, recent bioactive studies of LMF, and the pharmaceutical industry in general. Because of that increased demand, we tested the toxicity of LMF prepared from *L. japonica* by enzyme hydrolysis. The tests included bacterial reverse mutation assay, chromosome aberration assay, in vivo mouse micronucleus assay, and in vivo rat repeated dose 28-day oral toxicity assay.

## 2. Results and Discussion

### 2.1. Total Arsenic and Inorganic Arsenic Content of Laminaria japonica and LMF-LJ

Arsenic is one of the five identified industrial metals with strong neurotoxicity, the inorganic forms of AsIII and AsV are highly toxic, and the organic forms have varying degrees of toxicity [23]. Epidemiological studies have indicated that there are significant dose-response relationships between inorganic arsenic ingestion and cancer incidences [24,25]. In addition, organic arsenic has been revealed as a clastogenic agent in vitro [26] and a promoter of carcinogenesis in vivo [22]. We therefore carefully investigated the total arsenic content and arsenic species in raw *L. japonica* and LMF.

The total arsenic in the *L. japonica* of our study was 61.100 ± 3.110 mg/kg, a slightly higher value than the 30–54 mg/kg reported by [27]. It is believed that the seaweed accumulates arsenic from seawater and the arsenic concentration in the seaweed is related to the environmental conditions, growth, and metabolic rate [27]. According to the LC-ICP-MS results, the concentrations of the species of arsenic in *L. japonica* were as follows: AsB (34.31 ± 1.21 mg/kg) > MMA (9.27 ± 0.96 mg/kg) > DMA (9.23 ± 0.83 mg/kg) > AsC (59.00 ± 1.65 mg/kg). The species AsIII and AsV were not detected. The total arsenic in LMF-LJ was 6.200 ± 2.005 mg/kg, which showed a significant reduction in the concentration of organic arsenic (89.85%), and AsIII and AsV were not detected either (Table 1). Fortunately, no inorganic arsenic was detected in *L. japonica* and LMF-LJ. This is because the bioaccessibility of inorganic arsenic significantly increases after processing [19], which represents a toxicological risk of seaweed products. Reduction of organic arsenic is also an important issue for food safety, however, since significant liver tumor induction has been observed in rats that were treated with 200 ppm or more DMA [22].

International limits on inorganic arsenic content have been adopted to protect people. The concentration of inorganic arsenic in LMF-LJ measured in this study (<0.02 mg/kg) was in line with Taiwan (<1 mg/kg) [28], China (<1.5 mg/kg) [29], Australia (<1 mg/kg) [30], New Zealand (<1 mg/kg) [30], and the USA (<3 mg/kg) [31]. It is therefore suggested that the total arsenic content of the experimental LMF-LJ carried no toxicological concern.

### 2.2. Bacterial Reverse Mutation Assay

The bacterial reverse mutation assay is a widely employed method that uses bacteria to test whether a given chemical can cause mutations in the DNA of the test organism. Li et al. have demonstrated the toxicity of fucoidan from *L. japonica* in vivo but none have tested its genotoxicity in vitro [18]. We therefore tested, for the first time, the genotoxicity of LMF-LJ. No dose-dependent effect in revertant colonies was observed at LMF-LJ levels up to 5000 μg/mL. LMF-LJ did not cause more than a twofold increase in revertants per plate with or without S9 compared to the negative control. The positive control for each strain resulted in the expected significant increase in the number of revertant colonies (Table 2). Our data indicated no evidence of mutagenic potential under the conditions used in this assay for LMF-LJ. It has been reported that fucoidan and LMF of *Undaria pinnatifida* showed no mutagenicity up to 5000 μg/mL in the Ames test [32,33], results similar to those in our study.

### 2.3. Chromosome Aberration Assay

LMF-LJ gene mutagenicity was determined with the chromosome aberration assay. The highest dose tested in the chromosome aberration assay was 5000 μg/mL. No increases in structural or numerical chromosomal aberrations were observed at any dose of LMF-LJ (312.5–5000 μg/mL) with or without S9 compared to the negative control. The positive control, MMC, with or without S9, increased the frequency of cells with >10% chromosome aberration (Table 3). LMF-LJ therefore does not induce chromosome aberrations according to our study, as with LMF of *U. pinnatifida* [33].

## 3. Materials and Methods

### 3.1. Low Molecular Weight Fucoidan

The LMF from *L. japonica* (LMF-LJ) named Hi-Q Oligo-fucoidan^®^, was provided by Hi-Q Marine Biotech International Ltd. (New Taipei City, Taiwan). It was obtained by enzyme hydrolysis of the original fucoidan. The characteristics of LMF-LJ were as follows: average molecular weight of <667 Da with a 85.9% fucose content (127.2 ± 1.3 μmol/g), sulfate content 28.4% ± 2.1% (*w*/*w*), protein content 4.3% ± 0.3% (*w*/*w*), fat content 0.6% ± 0.1% (*w*/*w*), ash 4.1% ± 0.1% (*w*/*w*), and moisture content 3.9% ± 0.8% (*w*/*w*). The LMF-LJ was a light brownish-white powder and well soluble below the highest concentration.

### 3.2. Determination of Total Arsenic and Inorganic Arsenic Species

The methodology for the determination of total arsenic and inorganic arsenic species followed that of [21]. Briefly, milled samples of *L. japonica* and LMF-LJ were digested using an ETHOS 1 laboratory microwave system (Milestone, Leutkirch, Germany). Total arsenic determinations were carried out with an Agilent 7700e ICP-MS (Agilent Technologies, Santa Clara, CA, USA) with a microflow nebulizer; the detection limit was 0.010 mg/kg. LC-ICP-MS was used for the determination of arsenic species in sample extracts; the detection limit was 0.02 mg/kg. An Agilent 1260 Infinity Quaternary LC System (Agilent Technologies, Waldbronn, Germany) consisting of a solvent degassing unit, a binary pump, an autosampler, and a thermostatted column compartment was used. The 10 μ Hamilton PRP-X100 column (250 mm × 4.1 mm) was protected by a guard column filled with the same stationary phase. The outlet of the LC column was connected via PEEK capillary tubing (0.125 mm i.d.) to the nebulizer, which served as the arsenic-selective detector. The stock solutions of arsenic compounds were prepared from AsIII oxide (Sigma Aldrich, St. Louis, MO, USA) by dissolution in 0.2% NaOH and from AsV oxide hydrate (Sigma Aldrich, St. Louis, MO, USA). MMA, DMA, AsB, and AsC were purchased from Arcos-Organics (Fair Lawn, NJ, USA).

### 3.3. Bacterial Reverse Mutation Assay

The bacterial reverse mutation assay was conducted by the pre-incubation method in the presence and absence of S9 metabolic activation [34,35]. Tester strains included *Salmonella typhimurium* TA97a, TA98, TA100, TA102, and TA1535 with and without S9 and corresponding positive control agents such as 4-nitro-o-phenylenediamine (NPD), NaN_3_, mitomycin C (MMC), 2-aminofluorene (2-AF), benzo[α]pyrene (BP), and 2-aminoanthracene (2-AA). LMF and positive controls were dissolved in DMSO as a vehicle. A preliminary range-finding study was conducted for all tester strains at LMF-LJ concentrations of 100, 500, 1000, and 5000 μg/plate. Results of that study indicated the definitive study concentrations of 312.5, 625, 1250, 2500, and 5000 μg LMF-LJ /plate. For each treatment, 0.1 mL of LMF-LJ or control preparation was introduced into a sterilized test tube, to which 0.1 mL of bacterial suspension was added. For preparations with S9, 0.5 mL of S9 mix was also added; for preparations without S9, 0.5 mL of 0.1 M sodium phosphate buffer solution was added. Mixtures were then incubated with gentle shaking for 20 min at 37 °C, 120 rpm. After incubation, 4 mL of top agar (45 °C) containing 0.5 mm histidine/biotin was added, and then all substances were spread evenly on minimal glucose agar plates. After the top agar solidified, the plates were inverted and incubated for 48 h at 37 °C. The numbers of revertant colonies were counted by either an automatic colony analyzer or manual counting. The test substance was judged positive for mutagenicity when (1) substances induced a dose-dependent increase in the number of revertant colonies to a level greater than twofold of the negative control value and (2) the dose-dependent increase was reproducible.

### 3.4. Chromosome Aberration Assay

The experiment was carried out in triplicate to determine the effects of LMF-LJ (at concentrations of 312.5, 625, 1250, 2500, and 5000 μg/mL) on the induction of chromosomal aberrations in Chinese hamster ovary cells (CHO-K1, CCRC 60006). Cells were cultured in reconstituted minimum essential medium supplemented with 2.2 g sodium bicarbonate, 292 mg l-glutamine, streptomycin sulfate (100 μg/mL), penicillin G-Na (105 units), and 10% (*v*/*v*) fetal bovine serum (FBS) per liter. The cells were grown as monolayers in culture flasks and incubated in a humidified atmosphere of 5% CO_2_ in air at 37 °C [36].

The LMF-LJ was added after 1 h incubation in 4.0 mL of fresh culture medium with or without S9. The negative control was treated with FBS only; 2 μM mitomycin C was used as a positive control. After 22 h, 5 mg/mL colchicine was added for 2 h. Chromosomes were prepared according to standard procedures. Hypotonic treatment with 0.4% KCl (37 °C) was applied for 20 min. The cells were fixed with methanol and acetic acid (3:1) and the fixative was changed twice. Air-dried slides were stained with Giemsa (5%) and scored for chromosome aberrations according to [37]. The structural aberrations recorded were chromosome gap, chromatid gap, chromosome break, chromosome deletion, chromatid break, chromatid deletion, triradial, quadriradial, ring, complex rearrangement, dicentric, polyploidy, and pulverized cell. Each slide was scanned systematically and each set of metaphases was examined under 1000× magnification. After each type of aberration was recorded, the number of aberrant metaphases and total aberrations were calculated.

### 3.5. In Vivo Mouse Micronucleus Assay

Some 25 healthy male Imprinting Control Region (ICR) mice (six weeks of age) were used in this study; they were purchased from National Taiwan University College of Medicine Laboratory Animal Center (Taipei, Taiwan). All animals were seven weeks of age at the start of the experiment and were housed in a normal, environmentally controlled animal room with free access to pathogen-free feed and water *ad libitum*. Over three consecutive days, the mice were treated by gavage with 500, 1000, and 2000 mg/kg body weight (BW) LMF-LJ dissolved in saline. The negative control (0 mg/kg BW LMF-LJ) mice were treated identically with equal volumes of normal saline also via gavage throughout the study. MMC (2 mg/kg, i.p.) was administered as a positive control.

Whole blood smears were collected on the day following the last LMF-LJ administration or one day after MMC treatment. Whole blood smears were prepared on clean microscope slides, air dried, fixed in methanol, and stained with 1% brilliant cresyl blue (BCB) (Lot No. MKBH8545V, Sigma, St. Louis, MO, USA) for 10 min just before the evaluation with a fluorescence microscope (Nikon ECLIPSE E600, Tokyo, Japan). The frequency of reticulocytes (RETs) per total erythrocytes was determined using a sample size of 1000 erythrocytes per animal. The number of micronuclei (MNs) was determined using 2000 RET per animal. Briefly, immature erythrocytes (i.e., RETs) were identified by their orange-red color; MNs were identified by their yellow-green color (Kirkland, 1994).

All experimental procedures, including the use of experimental animals, were approved by Institutional Animal Care and Use Committee (IACUC), Chinese Culture University, Taiwan, Republic of China with permission number CCU-IACUC-104011 and CCU-IACUC-10509.

### 3.6. In Vivo Rat Repeated-Dose 28-Day Oral Toxicity Assay

40 male and 40 female Sprague-Dawley (SD) rats (four weeks of age) were used in this study; they were purchased from National Taiwan University College of Medicine Laboratory Animal Center (Taipei, Taiwan). All animals were six weeks of age at the start of the experiment and were housed in a normal, environmentally controlled animal room with free access to pathogen-free feed and water *ad libitum*. These 80 SD rats were randomly divided into a control and three dose levels (500, 1000, and 2000 mg/kg BW LMF-LJ) with 10 males and 10 females in each group. Concentrations of 500, 1000, and 2000 mg/kg BW LMF-LJ were dissolved in saline and then administered by oral gavage in 10 mL/kg of BW on a daily basis for 28 days. The control (0 mg/kg BW LMF-LJ) rats were treated identically with equal volumes of normal saline also via gavage throughout the study. Rats were anesthetized with diethyl ether followed by cervical decapitation. Blood samples were collected for evaluation of clinical hematology and biochemistry [38,39].

#### 3.6.1. Body Weight, Food Intake, and Water Consumption

Body weights and food and water intake were measured at 0 days before treatment and at 7, 14, and 28 days after treatment. Food and water intake was measured in mg/kg BW/day; the amount of food and water was measured before supplying them to the cage and the remainder was measured the next day.

#### 3.6.2. Observation of Clinical Signs

All abnormal clinical signs were noted and measured before and after dosing, at least twice a day based on the functional observational battery test.

#### 3.6.3. Urinalysis

Before sacrifice, a 16-h (17:00 to next day 9:00) urine sample was collected and the urine parameters were determined by a Clinitek 500 urine chemistry analyzer (Bayer Health Care, Cambridge, MA, USA) and a Multistix 10SG reagent strip (Bayer, Elkhart, IN, USA).

#### 3.6.4. Hematology

A hematological examination was conducted at the end of the study. Hematological parameters were determined by an automated hematology analyzer (XT-1800i, Sysmex Corporation, Kobe, Japan). Prothrombin time (PT) and activated partial thromboplastin time (APTT) were determined by an automated coagulation analyzer (CA-1500, Sysmex Corporation, Kanagawa, Japan).

#### 3.6.5. Serum Biochemistry

The animals fasted for more than 8 h before being sacrificed. Blood was collected and centrifuged at 1500× *g* for 15 min to collect serum. Serum was then analyzed by an automated biochemistry analyzer (Vitros 5.1 FS, Johnson & Johnson, New Brunswick, NJ, USA) for serum biochemistry parameters such as alanine aminotransferase, aspartate aminotransferase, alkaline phosphatase, total bilirubin, total protein, albumin, globulin, blood urea, nitrogen, creatinine, total cholesterol, triglyceride, prothrombin time, and activated partial thromboplastin time.

#### 3.6.6. Organ Weight

All organs were carefully examined macroscopically and the brain, heart, kidneys, liver, spleen, adrenals, testes (males), and ovaries (females) were weighed relative to total body weight.

#### 3.6.7. Histopathology

All organs were fixed in 10% neutral buffered formalin solution for pathologic examination. The organs from the control and test groups were further processed, embedded in paraffin, sectioned at 2 μm by microtome (Finesse 325, Thermo Shandon Ltd., Cheshire, UK), stained with hematoxylin and eosin (H&E), and evaluated for histopathology under a microscope (BX-51, Olympus, Tokyo, Japan).

### 3.7. Statistical Analysis

Numerical data are presented as means ± standard deviation. The data was analyzed by a one-way analysis of variance (ANOVA), which was followed by the least significant difference test using SPSS (Chicago, IL, USA) version 10 software. A *p*-value of <0.05 was considered a significant difference.

## 4. In Vivo Mouse Micronucleus Assay

The micronucleus test is a mammalian in vivo test that detects damage of the chromosomes or mitotic apparatus by chemicals. It is based on an increase in the frequency of micronucleated polychromatic erythrocytes in the bone marrow of treated animals. No significant clinical symptoms were observed during the experiment, and no mouse died.

LMF-LJ administration at 500, 1000, and 2000 mg/kg BW caused no significant change in RETs/1000 erythrocytes% and MNs/2000 RETs%, while the positive control, MMC, significantly changed in RETs/1000 erythrocytes% and MNs/2000 RETs%, as expected (Table 4). None of the fucoidan or LMF levels changed significantly in the micronucleus assay [38,39,40]. These results suggested that the oral administration of LMF-LJ did not disrupt the normal formation of erythrocytes and that the intake of less than 2000 mg/kg BW was safe.

## 5. In Vivo Rat Repeated Dose 28-Day Oral Toxicity Assay

### 5.1. Body Weights, Food Intake, Water Consumption, and Clinical Signs

**Figure 1:**
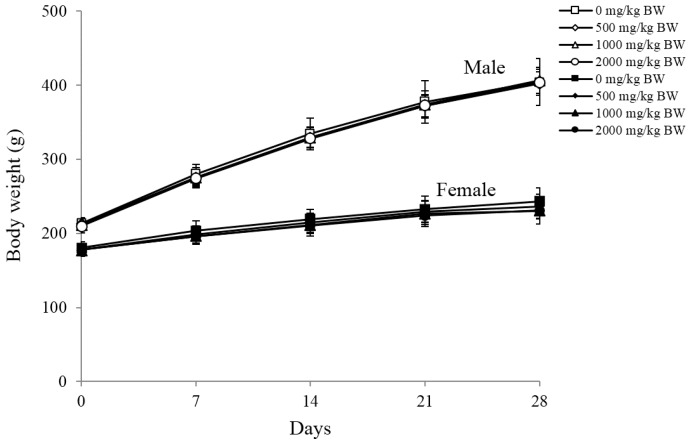
Growth curves for male (open symbols) and female (solid symbols) rats treated with LMF-LJ for 28 days. Values were expressed as mean ± SD, *n* = 10.

The Organisation for Economic Co-operation Development (OECD) (2001)-recommended guideline for the highest dose of test material is 2000 mg/kg BW; the highest dose of LMF-LJ was therefore selected as 2000 mg/kg, which was repeated daily for the 28-day oral toxicity assay [41]. No significant changes in body weight, food intake, or water intake were detected in all groups (500, 1000, and 2000 mg/kg BW) tested compared to the normal saline control (Figure 1). No significant clinical symptoms were observed during the experiment, and no rat died.

### 5.2. Urinalysis Results

The urinary volume of the control rats was 6.3 ± 0.3 mL/16 h in the male groups and 5.9 ± 0.8 mL/16 h in the female groups; there was no significant increase in urine production after treating with LMF-LJ. There was no significant difference between dosed groups and normal saline control in levels of urine SG, pH, protein, Uro, and ketone; Glu and Nit were absent in the urine of all groups. Although there were trace levels of Oc. blood in 2000 mg/kg BW of only one mouse in the male groups, there were no significant abnormalities to note (Table 5). Rats that received fucoidan of *U. pinnatifida* for 28 days also showed 80 Ery/μL in urine [27]. This variation was therefore considered within the normal physiological changes for rats and not a dose-related effect.

### 5.3. Hematological, Blood Clotting, and Serum Biochemistry Results

LMF-LJ did not increase the activity of serum toxicity marker enzymes (alanine aminotransferase (ALT), aspartate aminotransferase (AST)) up to 2000 mg/kg BW, indicating normal liver function. LMF-LJ administration also did not affect hematological parameters (red blood cell (RBC), white blood cell (WBC), platelet count (PLK), neutrophil (NEUT), lymphocyte (LYMPH)), blood clotting time (prothrombin time (PT), activated partial thromboplastin time (APTT)), and some serum biochemical parameters (alkaline phosphatase (ALP), total bilirubin (T-BIL), total protein (TP), albumin (ALB), globulin (GLO), blood urea nitrogen (BUN), total cholesterol (TC), Na, K, Ca, and P), but creatinine (CRE) and triglyceride (TG) showed significant decreases compared to the control (Table 6). Creatinine levels fluctuated after treatment with different concentrations of fucoidan from *Cladosiphon okamuranus* in Wistar rats [18,42]. In another study, creatinine levels were reduced in a male rat but increased in a female one when treated with fucoidan from *Undaria pinnatifida.* That may suggest that the fucoidan extract from different sources may have different effects on creatinine levels [27]. In agreement with those studies, the fucoidan extract from *Laminaria japonica* also correlated with decreased plasma creatinine levels in the Active Heymann Nephritis rat model [43]. From our findings, the fucoidan extract from *L. japonica* reduced creatinine levels in male and female rats. These results are consistent with previously reports [27].

Triglyceride level has been suggested as causal factor for cardiovascular disease and type 2 diabetes mellitus [44,45]. Fucoidan polysaccharide sulfuric acid ester extract from *Laminaria japonica* at concentrations ranging from 0.1 to 0.4 g/kg has been known to significantly reduce total serum triglycerides in hyperlipidemic rats [46]. Fucoidan extract from *Cladosiphon okamuranu* (150 mg/kg/day for seven days) can reduce the triglyceride level in myocardial infarction rats [47]. Fucoidan from *Undaria pinnatifida* (concentrations ranging from 150 to 1350 mg/kg) has been correlated with an increased level of triglycerides in male rats and a decreased level of triglycerides in female rats relative to controls. In our study, we found that fucoidan from *L. japonica* correlated with an increased level of triglycerides in male rats and a decreased level in female rats, which is consistent with the previous study [46].

Although there were significant changes in CRE, the values remained within the normal range (0.4–1.4 mg/dL) [48]. Similar effects were also observed by [27] and [49], namely that fucoidan can decrease CRE slightly in serum. LMF-LJ significantly reduced TG in serum; this might have been caused by an increase in levels of lipid metabolizing enzymes [46,50]. Li et al. studied fucoidan from *L. japonica* in Wistar rats for six months and reported no significant toxicological changes with 300 mg/kg BW/day, though prolonged clotting times were seen at doses of 900 and 2500 mg/kg BW/day [18]. Gideon and Rengasamy demonstrated similar results in prolonged clotting times when Wistar rats received 1500 mg/kg BW fucoidan from *Cladosiphon okamuranus* [42]. This phenomenon, however, was not observed in our study.

### 5.4. Organ Weight and Histopathological Results

Absolute and relative organ weights of male and female rats are summarized in Table 7. There were no meaningful changes in the gross findings of eight principal organs in all experimental groups. No histopathological results were observed in any of the LMF-LJ experimental groups (data not shown).

## 6. Conclusions

In conclusion, LMF-LJ at 5000 μg/mL displayed no mutagenicity by either the bacterial reverse mutation or the chromosomal aberration assay in vitro. Moreover, LMF-LJ caused no formatting disruption of erythrocytes in vivo. Through 28 days of repeated oral administration to SD rats, it was found that up to 2000 mg/kg BW/day of LMF-LJ caused no toxicological indications. The use of LMF-LJ is presently expected to be safe and may prove to be a useful bioactive agent after further toxicity research.

## Figures and Tables

**Table 1:** Total arsenic and inorganic arsenic content of *Laminaria japonica* and LMF-LJ.

Species	*Laminaria japonica* (mg/kg)	LMF-LJ (mg/kg)
AsIII	ND ^a^	ND
AsV	ND	ND
MMA	9.27 ± 0.96	1.35 ± 0.63
DMA	9.23 ± 0.83	ND
AsB	34.31 ± 1.21	4.77 ± 0.88
AsC	6.19 ± 2.17	ND
Total arsenic (sum)	59.00 ± 1.65	6.12 ± 2.14
Total arsenic (direct)	61.100 ± 3.110	6.200 ± 2.005

^a^ Detection limit was 0.02 ppm. LMF-LJ: low molecular weight fucoidan from *L. japonica*; ASIII: arsenite; ASV: arsenate; MMA: monomethylarsonic acid; DMA: dimethlarsinic acid; AsB: arsenobetaine; AsC: arsenocholine; ND: Not detected.

**Table 2:** Results of the definitive bacterial reverse mutation assay on LMF-LJ.

LMF-LJ (μg/mL)	S9	Average Number of Revertants (Number of Colonies/Plate)				
		Frameshift		Base Pair Substitution		Transition
		TA97a	TA98	TA100	TA1535	TA102
Negative control	–	109.7 ± 7.2 ^a^	17.3 ± 2.9	102.0 ± 9.5	11.7 ± 1.5	475.0 ± 27.7
312.5	–	111.0 ± 5.5	14.3 ± 0.6	101.7 ± 11.5	12.3 ± 0.5	536.0 ± 31.4
625	–	123.3 ± 13.5	16.3 ± 4.5	101.7 ± 8.3	13.0 ± 2.6	541.3 ± 38.4
1250	–	128.3 ± 11.6	15.3 ± 5.8	109.0 ± 6.9	12.0 ± 3.0	494.7 ± 37.4
2500	–	114.0 ± 13.0	9.7 ± 1.5	103.0 ± 11.5	11.0 ± 3.0	480.7 ± 56.1
5000	–	112.0 ± 7.0	14.3 ± 1.1	104.0 ± 2.6	14.0 ± 3.0	432.0 ± 31.4
Positive control						
NPD	–	524.7 ± 104.5 *	836.0 ± 72.6 *			
NaN_3_	–			1209.7 ± 263.3 *	352.3 ± 45.6 *	
MMC	–					1970.7 ± 113.4 *
Negative control	+	138.3 ± 7.3	24.7 ± 1.5	122.0 ± 20.8	11.7 ± 1.5	503.0 ± 70.5
312.5	+	128.3 ± 16.0	27.0 ± 2.0	105.3 ± 8.1	12.0 ± 2.6	538.0 ± 14.0
625	+	136.0 ± 15.6	18.0 ± 4.3	123.0 ± 5.0	11.3 ± 4.6	516.7 ± 36.0
1250	+	152.3 ± 6.6	23.7 ± 1.5	121.7 ± 8.5	13.7 ± 0.5	510.7 ± 32.3
2500	+	123.3 ± 11.1	24.0 ± 3.4	108.0 ± 11.7	12.7 ± 3.2	485.3 ± 19.4
5000	+	128.0 ± 11.5	17.3 ± 4.9	111.3 ± 15.3	15.3 ± 2.5	452.0 ± 26.0
Positive control						
2-AF	+	367.3 ± 28.3 *		287.3 ± 22.6		
BP	+		59.7 ± 10.2 *			
2-AA	+				108.7 ± 15.5 *	1009.3 ± 56.05 *

^a^ Values were expressed as mean ± SD, *n* = 3; * The number of revertant colonies was two-fold greater than the negative control. NPD: 4-nitro-o-phenylenediamine; MMC: mitomycin C; 2-AF: 2-aminofluorene; BP: benzo[α]pyrene; 2-AA: 2-aminoanthracene.

**Table 3:** Results of chromosomal aberrations in CHO-K1 cells after 3 h treated with LMF-LJ, absence and non-absence of S9.

LMF-LJ (μg/mL)	S9	Cell Viability (×10^6^ cells)	Number of Aberrations													Aberrant Cell (% ± SD) ^a^
			SG	TG	SB	SD	TB	TD	TR	QR	R	CR	DC	PP	PC	
Negative control ^b^	–	3.45 ± 0.02	0	0	0	0	0	0	0	0	0	0	0	0	0	0.0
Positive control ^c^	–	2.78 ± 0.09	0	0	0	0	2	0	8	2	0	0	0	0	0	12.6 ± 1.1 *
312.5	–	3.58 ± 0.01	0	0	0	0	0	0	0	0	0	0	0	0	0	0.0
625	–	3.70 ± 0.01	0	0	0	0	0	0	0	0	0	0	0	0	0	0.0
1250	–	3.85 ± 0.03	0	0	0	0	0	0	0	0	0	0	0	0	0	0.0
2500	–	3.63 ± 0.02	0	0	1	0	0	0	0	0	0	0	0	0	0	1.4 ± 0.5
5000	–	3.53 ± 0.04	0	0	0	0	0	0	0	0	0	0	0	0	0	0.0
Negative control	+	3.68 ± 0.03	0	0	0	0	0	0	0	0	0	0	0	0	0	0.0
Positive control	+	2.60 ± 0.12	0	0	0	0	7	0	7	5	0	0	0	0	2	21.3 ± 1.4 *
312.5	+	3.90 ± 0.01	0	0	0	0	0	0	0	0	0	0	0	0	0	0.0
625	+	3.73 ± 0.04	0	0	0	0	0	0	0	0	0	0	0	0	0	0.0
1250	+	3.88 ± 0.02	0	0	0	0	0	0	0	0	0	0	0	0	0	0.0
2500	+	3.58 ± 0.02	0	0	0	0	0	0	0	1	0	0	0	0	0	1.2 ± 0.3
5000	+	3.80 ± 0.01	0	0	0	0	0	0	0	0	0	0	0	1	0	1.5 ± 1.0

^a^ Frequency of aberrant cells in 100 mataphases scored; ^b^ Culture medium with FBS; ^c^ 2 μM MMC was used as a mutagen; * *p* < 0.05 as compared with negative control; SG: chromosome gap; TG: chromatid gap; SB: chromosome break; SD: chromosome deletion; TB: chromatid break; TD: chromatid deletion; TR: triradial; QR: quadriradial; R: ring; CR: complex rearrangement; DC: dicentric; PP: polyploidy; PC: pulverized cell.

**Table 4:** Results of micronucleus assay in peripheral blood erythrocytes of mice treated with LMF-LJ.

Sample	Dose (mg/kg BW)	Body Weight (g)		RETs/1000 Erythrocytes (%)	MNs/2000 RET (%)	Clinical Signs	Mortalities (Dead/Total)
		First treatment	Sacrifice			ND	0/5
Negative control		31.44 ± 1.49 ^a^	30.5 ± 1.9	30.5 ± 1.9	1.2 ± 0.8	ND	0/5
Positive control ^b^	1	30.50 ± 1.76	10.9 ± 4.6 *	10.9 ± 4.6 *	29.3 ± 5.4 *	ND	0/5
LMF	500	31.70 ± 1.56	26.4 ± 3.2	26.4 ± 3.2	1.0 ± 0.7	ND	0/5
	1000	31.64 ± 1.80	24.8 ± 3.9	24.8 ± 3.9	1.8 ± 1.3	ND	0/5
	2000	32.12 ± 1.43	28.5 ± 3.6	28.5 ± 3.6	1.4 ± 1.5	ND	0/5

^a^ Values were expressed as mean ± SD, *n* = 5; ^b^ MMC was used as a mutagen; * *p* < 0.05 as compared with negative control; RETs: reticulocytes, MNs: micronucleus.

**Table 5:** Urinalysis results of male and female rats treated with LMF-LJ for 28 days.

Sex	Dose (mg/kg BW)	Volume (mL)	SG	pH	Protein (mg/dL)	Uro (EU/dL)	Glu	Bilirubin		Ketone ^a^		Nit	Oc. Blood	
							N	N	±	+1	+2	N	N	±
Male	0	6.7 ± 0.3 ^b^	1.024 ± 0.001	7.1 ± 0.1	86.2 ± 8.3	0.32 ± 0.03	10 ^c^	5	5	2	8	10	10	0
	500	6.7 ± 0.8	1.019 ± 0.004	7.2 ± 0.4	97.7 ± 10.2	0.28 ± 0.02	10	4	6	3	7	10	10	0
	1000	6.4 ± 0.6	1.025 ± 0.002	7.0 ± 0.2	92.4 ± 9.6	0.30 ± 0.03	10	5	5	2	8	10	10	0
	2000	6.8 ± 0.2	1.024 ± 0.006	6.9 ± 0.1	98.1 ± 11.4	0.30 ± 0.01	10	5	5	1	9	10	9	1
Female	0	5.9 ± 0.8	1.019 ± 0.005	7.2 ± 0.6	75.6 ± 6.5	0.26 ± 0.01	10	7	3	8	2	10	10	0
	500	6.5 ± 0.3	1.021 ± 0.003	7.1 ± 0.4	83.1 ± 8.9	0.31 ± 0.08	10	6	4	3	7	10	10	0
	1000	5.8 ± 0.7	1.022 ± 0.007	6.8 ± 0.3	79.5 ± 10.0	0.30 ± 0.01	10	9	1	7	3	10	10	0
	2000	6.2 ± 0.4	1.027 ± 0.006	6.9 ± 0.2	89.2 ± 7.8	0.29 ± 0.02	10	7	3	6	4	10	10	0

^a^ +1: 5–15 mmol/l; +2: 15–30 mmol/l; ^b^ Values were expressed as mean ± SD, *n* = 10; ^c^ Number of rats with each result; SG: specific gravity; Uro: urobilinogen; Glu: glucose; Nit: nitrite; Oc. blood: occult blood; N: negative; ±: trace.

**Table 6:** Hematological, blood clotting and serum biochemical results of male and female rats treated with LMF-LJ for 28 days.

Sex	Male				Female			
Dose (mg/kg BW)	0	500	1000	2000	0	500	1000	2000
RBC (M/μL)	7.82 ± 0.19 ^a^	7.59 ± 0.26	7.87 ± 0.35	7.70 ± 0.15	7.90 ± 0.31	8.08 ± 0.34	8.10 ± 0.56	8.16 ± 0.55
WBC (K/μL)	8.96 ± 1.51	9.61 ± 0.94	10.43 ± 1.99	9.51 ± 1.38	6.85 ± 0.99	6.44 ± 1.97	6.04 ± 2.57	6.49 ± 1.68
PLT (K/μL)	1050.2 ± 66.7	1043.0 ± 102.0	1030.9 ± 93.8	957.9 ± 61.2	1025.9 ± 87.0	976.0 ± 108.9	1039.5 ± 77.2	1050.1 ± 109.1
NEUT (%)	16.97 ± 2.44	15.97 ± 5.30	14.29 ± 5.00	17.89 ± 5.58	14.82 ± 3.75	11.70 ± 4.57	10.11 ± 3.73	15.80 ± 6.70
LYMPH (%)	77.85 ± 2.75	80.17 ± 5.59	81.55 ± 5.44	76.90 ± 5.88	79.83 ± 3.97	80.17 ± 4.77	81.91 ± 3.67	79.87 ± 8.18
PT (sec)	14.12 ± 1.84	12.77 ± 0.97	13.46 ± 1.42	12.94 ± 0.97	9.97 ± 0.27	9.91 ± 0.12	9.84 ± 0.38	9.85 ± 0.14
APTT (sec)	18.13 ± 1.39	16.91 ± 1.06	17.29 ± 1.22	17.48 ± 1.01	15.33 ± 0.66	15.56 ± 0.84	16.35 ± 2.25	15.54 ± 0.27
ALT (U/L)	31.0 ± 4.1	32.5 ± 5.3	35.2 ± 8.5	35.8 ± 8.1	26.1 ± 3.0	29.1 ± 8.2	27.1 ± 6.6	31.0 ± 5.7
AST (U/L)	102.9 ± 17.6	106.3 ± 9.3	104.0 ± 17.3	118.3 ± 12.8	148.9 ± 21.7	130.5 ± 21.9	138.5 ± 21.3	146.7 ± 19.6
ALP (U/L)	171.6 ± 34.8	177.1 ± 28.6	181.2 ± 32.9	184.5 ± 25.2	98.4 ± 20.8	102.4 ± 25.8	101.3 ± 19.7	109.6 ± 21.02
T-BIL (mg/dL)	0.055 ± 0.016	0.045 ± 0.016	0.05.0 ± 0.000	0.05.0 ± 0.000	0.05.0 ± 0.000	0.05.0 ± 0.000	0.055 ± 0.016	0.05.0 ± 0.000
TP (g/dL)	6.46 ± 0.34	6.24 ± 0.30	6.46 ± 0.28	6.30 ± 0.24	6.93 ± 0.38	7.12 ± 0.46	6.90 ± 0.39	6.89 ± 0.62
ALB (g/dL)	4.12 ± 0.20	3.97 ± 0.13	4.04 ± 0.15	3.98 ± 0.14	4.42 ± 0.24	4.56 ± 0.28	4.38 ± 0.29	4.38 ± 0.35
GLO (g/dL)	2.34 ± 0.22	2.27 ± 0.18	2.42 ± 0.16	2.32 ± 0.13	2.51 ± 0.17	2.56 ± 0.18	2.52 ± 0.21	2.51 ± 0.29
BUN (mg/dL)	15.26 ± 2.42	15.00 ± 1.86	15.05 ± 1.18	15.57 ± 1.58	17.43 ± 2.77	16.45 ± 2.25	17.36 ± 2.64	17.60 ± 3.31
CRE (mg/dL)	0.54 ± 0.09	0.47 ± 0.02 *	0.47 ± 0.05 *	0.50 ± 0.04	0.62 ± 0.06	0.53 ± 0.06 *	0.52 ± 0.04 *	0.55 ± 0.04 *
TC (mg/dL)	64.2 ± 13.9	66.3 ± 13.0	56.4 ± 7.4	61.5 ± 7.7	77.4 ± 15.6	74.7 ± 15.0	84.4 ± 19.5	67.8 ± 12.1
TG (mg/dL)	53.5 ± 10.4	37.9 ± 9.5 *	35.8 ± 6.9 *	41.4 ± 8.1 *	46.4 ± 8.1	37.7 ± 13.4 *	37.6 ± 10.8 *	31.3 ± 9.4 *
Na (mmol/L)	147.4 ± 1.0	146.2 ± 1.3	147.2 ± 1.5	147.2 ± 1.0	145.1 ± 1.4	145.7 ± 2.0	144.6 ± 2.1	146.1 ± 1.5
K (mmol/L)	7.21 ± 1.07	7.83 ± 0.71	7.71 ± 0.70	7.01 ± 1.06	7.38 ± 0.64	7.33 ± 0.51	7.23 ± 0.89	7.34 ± 1.36
Ca (mg/dL)	10.80 ± 0.36	11.08 ± 0.23	11.21 ± 0.50	11.15 ± 0.36	11.38 ± 0.39	11.67 ± 0.27	11.32 ± 0.43	11.51 ± 0.59
P (mg/dL)	13.32 ± 2.79	13.70 ± 0.90	14.03 ± 1.30	14.04 ± 1.00	13.09 ± 1.11	12.65 ± 1.04	12.00 ± 0.85	13.87 ± 1.28

^a^ Values were expressed as mean ± SD, *n* = 10; * *p* < 0.05 as compared with negative control; RBC: red blood cell; WBC: white blood cell; PLT: platelet count; NEUT: neutrophil; LYMPH: lymphocyte; ALT: alanine aminotransferase; AST: aspartate aminotransferase; ALP: alkaline phosphatase; T-BIL: total bilirubin; TP: total protein; ALB: albumin; GLO: globulin; BUN: blood urea nitrogen; CRE: creatinine; TC: total cholesterol; TG: triglyceride; PT: prothrombin time; APTT: activated partial thromboplastin time.

**Table 7:** Absolute and relative organ weights of male and female rats treated with LMF-LJ for 28 days.

Sex		Male				Female			
Dose (mg/kg BW)		0	500	1000	2000	0	500	1000	2000
Brain	Weight (g)	1.99 ± 0.09 ^a^	1.95 ± 0.08	1.98 ± 0.07	1.95 ± 0.06	1.82 ± 0.07	1.75 ± 0.10	1.82 ± 0.05	1.81 ± 0.09
Heart	Weight (g)	1.39 ± 0.11	1.34 ± 0.07	1.36 ± 0.07	1.36 ± 0.09	0.81 ± 0.07	0.83 ± 0.08	0.82 ± 0.08	0.80 ± 0.03
	Ratio ^b^	0.69 ± 0.07	0.69 ± 0.03	0.69 ± 0.04	0.70 ± 0.05	0.44 ± 0.04	0.47 ± 0.03	0.45 ± 0.04	0.44 ± 0.02
Kidneys	Weight (g)	3.09 ± 0.23	2.99 ± 0.24	2.97 ± 0.25	3.06 ± 0.29	1.66 ± 0.06	1.66 ± 0.18	1.56 ± 0.14	1.66 ± 0.12
	Ratio	1.55 ± 0.14	1.52 ± 0.08	1.50 ± 0.16	1.57 ± 0.14	0.91 ± 0.05	0.95 ± 0.08	0.85 ± 0.07	0.92 ± 0.07
Liver	Weight (g)	13.24 ± 1.71	12.45 ± 1.19	12.82 ± 0.64	12.66 ± 0.64	6.97 ± 0.61	7.29 ± 0.76	7.23 ± 0.89	6.97 ± 0.55
	Ratio	6.65 ± 0.97	6.38 ± 0.67	6.48 ± 0.44	6.50 ± 0.39	3.82 ± 0.38	4.17 ± 0.39	3.96 ± 0.45	3.86 ± 0.34
Spleen	Weight (g)	0.72 ± 0.11	0.70 ± 0.08	0.71 ± 0.06	0.74 ± 0.11	0.43 ± 0.06	0.42 ± 0.06	0.49 ± 0.08	0.42 ± 0.05
	Ratio	0.36 ± 0.06	0.36 ± 0.04	0.36 ± 0.03	0.38 ± 0.06	0.23 ± 0.03	0.23 ± 0.02	0.26 ± 0.04	0.23 ± 0.02
Adrenals	Weight (g)	0.050 ± 0.006	0.053 ± 0.004	0.055 ± 0.005	0.050 ± 0.008	0.060 ± 0.006	0.059 ± 0.007	0.058 ± 0.008	0.059 ± 0.008
	Ratio (%) ^c^	2.514 ± 0.347	2.733 ± 0.225	2.785 ± 0.281	2.579 ± 0.410	3.320 ± 0.380	3.386 ± 0.385	3.223 ± 0.463	3.271 ± 0.511
Testes	Weight (g)	2.98 ± 0.16	3.13 ± 0.28	3.03 ± 0.31	2.92 ± 0.21	-	-	-	-
	Ratio	1.49 ± 0.09	1.60 ± 0.15	1.53 ± 0.19	1.50 ± 0.09	-	-	-	-
Ovaries	Weight (g)	-	-	-	-	0.067 ± 0.010	0.076 ± 0.008	0.074 ± 0.016	0.072 ± 0.013
	Ratio (%)	-	-	-	-	3.680 ± 0.471	4.397 ± 0.562	4.072 ± 0.886	4.031 ± 0.822

^a^ Values were expressed as mean ± SD, *n* = 10; ^b^ Organ weight/brain weight; ^c^ Organ weight/brain weight × 100%.
